# Family Self-Care in Chronic Disease Management: An Evolving Care Pattern?

**DOI:** 10.1177/23779608231226069

**Published:** 2024-02-04

**Authors:** Teresa Dionísio Mestre, Ermelinda Valente Caldeira, Manuel José Lopes

**Affiliations:** 1 Comprehensive Health Research Centre [CHRC], Évora, Portugal; 2 Health Department, Polytechnic Institute of Beja, Beja, Portugal; 3 Department of Nursing, University of Évora, Évora, Portugal

**Keywords:** chronic disease, family nursing, family self-care, nursing, self-care

## Abstract

**Introduction:**

Self-care emerges in the family context, despite being seen as an individual determinant of health. The family, understood as a system and social unit, converges to a pattern of self-care and not to a sum of it, assuming relevance at certain moments of the life cycle, particularly in the management of chronic disease.

**Purpose:**

To perform the transposition of individual self-care to the family self-care, considering the current family's needs and characteristics, by adopting family self-care as the core concept of a care pattern as a determinant of family health.

**Conclusion:**

The family unit is the most influential factor in the health status of individuals, and it will be through family self-care behaviors that families can be healthier by managing their diseases more effectively. They seek to achieve family health, maintaining health through health promotion and disease management practices, always mediated by family self-care behaviors.

## Introduction

For several decades, holistic nursing care has been transmitted as a philosophy that guides not only person-centered care but also, particularly the family, as an entity inseparable from any individual, emerging from the concepts of humanism and holism (Jasemi et al., 2017). However, it is evident that, despite the contributions of various theoretical models for the evolution of knowledge that contextualizes the work of nurses with the family (Adamsons et al., 2022; Wright & Leahey, 2012), there remains a significant gap between them and effective practice (Duhamel et al., 2015). Apparently, family-centered care continues to be the expression of an ideal and not a prevalent praxis, not only in contexts of hospitalization, but also in the community (Duhamel et al., 2015).

If we go back in time and analyze the conceptual evolution of the family, we perceive that the notion of social unit and/or fundamental unit of society has been preserved from the perspective of several authors and theorists (Adamsons et al., 2022; Figueiredo, 2012; Gray, 1996; Kamaryati & Malathum, 2020). The structure of the family has undergone several changes; however, family roles have remained somewhat constant over time (Figueiredo, 2012; Park et al., 2018; Kamaryati & Malathum, 2020). Thus, in the present, it is important rather than focusing on a stable construct, to consider that the concept of family can take on different formats, depending on both the individual and family context, biological bonds, legal and/or religious status, among others. The economic, political, and sociocultural changes we are witnessing erect a diversity of family structures that hardly fit into a single definition (Figueiredo, 2012; Park et al., 2018). Whatever the type of family we find that all of them are organized through a structure of relationships where roles and functions are defined according to social expectations (Olabisi et al., 2023). Nevertheless, and to shape health and healthcare experiences, it is important for healthcare professionals and especially for family nurses to consider the ways that family structure and process interact within broader sociocultural contexts (Beckmeyer & Russell, 2022; Russell et al., 2022). Thus, it is essential to analyze individual behavior, but in the context in which it occurs, with the focus of analysis necessarily becoming systemic (Seltzer, 2019). We can therefore assume that the social determinants of health are fundamental when it comes to healthcare. These include culture, social norms, social policies, and political systems that have a direct impact on the health of families and, which to a certain extent, are influenced by families. In fulfilling the family functions of socializing and protecting their members, incorporate and interpret the wider sociocultural and political worlds for their members. Therefore, issues affecting any part of the ecological worlds of individuals can affect the health of families, potentially providing protection or increasing risk (Deatrick, 2017). In this sequence, the family translates a complex network of relationships and emotions, seen as a social unit (International Council of Nurses, 2012) or a collective whole, composed of members connected through consanguinity, emotional affinity, or legal kinship, including people who are important to the client, the target of care (Kamaryati & Malathum, 2020). Thus, it is essential to analyze individual behavior, but in the context in which it occurs, the focus of analysis becomes necessarily systemic.

In this perspective, if the family is understood as a system and social unit, the whole it represents will be greater than the sum of its parts (Adamsons et al., 2022) and self-care (SC), a basic dimension of it (Orem, 2001; World Health Organization [WHO], 2022). The SC exhibits individual competence to take care of oneself, is inherent in the human being and develops throughout the life cycle (Orem, 2001). Thus, it can be understood as a health resource, of extreme relevance to the well-being and quality of life in the family context. In this sense, the transposition of individual SC to the family seems extremely relevant, given the needs and characteristics of the family, through its adoption as a central concept of a pattern of care determinant of family health.

The interest in studying and developing nursing knowledge focused on the family, particularly in the idiosyncrasy of SC, is mainly related to the current and relevant epidemiological and sociofamily conjuncture.

Currently, the prevalence of chronic diseases leads the population context, thus nurses and other healthcare professionals need to have knowledge that allows them, in an integrated approach, care, and to teach the family as a fundamental unit of society, in the acquisition and maintenance of SC (Matthys et al., 2023; Miller & Reihlen, 2023; Tulu et al., 2021). Family care is widely considered a relational experience and takes place within a historical context of family interaction (Esandi et al., 2021). To this end, the concept of SC in its individual aspect and transposition to the family seems to converge with three theories: the nursing self-care theory of Orem (Orem, 2001), the middle range theory of self-care of chronic disease of Riegel (Riegel et al., 2012) and the systems general theory of von Bertalanffy (von Bertalanffy, 2015).

Therefore, the main objective of this article is to develop the concept that unites the family to SC and understand if there is a pattern or culture of family self-care (FSC), trying to explain how it can develop, that is, to demonstrate that SC being indispensable in the individual context, and the family the primordial unit where it develops, does not direct us to a sum of SC within this same family but eventually to a pattern of FSC, with relevance at certain key moments in the life cycle. This pattern, inherent in the family, conditions and is conditioned by each of its members.

## Background of Self-Care

Thinking about the SC refers us to a powerful concept in the context of health and particularly nursing as a discipline of care. This term is a central and transversal question in the life of all people, present in human life, and in their vital process (Mills et al., 2018). It aroused interest in the year 1948, at the Constitution Congress of the World Health Organization, in conceiving health as a “state of complete physical, mental and social well-being,” not only consisting of the absence of disease but recognizing the psychological and social components, as well as the interaction between these factors, attributing an important role to risk behaviors in the health context (WHO, 2022). Thus, the adoption of a biopsychosocial perspective was initiated, which pointed to an integral care of the person, directed to the promotion of self-responsibility in SC, as a resource to promote quality of life and well-being. However, its conceptualization was initiated by Dorothea Orem in 1956 and formally validated in 1967 through the work done by the Nursing Development Conference Group. Orem (2001) developed the nursing theory of self-care deficit that encompasses three interrelated theories, taking as central construct the term SC: the self-care theory, the theory of self-care deficit, and the theory of nursing systems. In the present context, the self-care theory may have a special relevance. In this conception, every person has the potential to self-care, by possessing skills, knowledge and experience acquired throughout life. And, in situations where SC supplants its ability to do so, it needs support from both people with social responsibilities (e.g., family, friends) and healthcare professionals (e.g., nurses). The basic premise of Orem's concept of SC is the individual responsibility to develop self-care measures, that is, the action of self-care. And an important result indicator is that the person can perform SC with scarce contacts to health services, allowing the family to exercise control over the environment and work to achieve pre-established health behaviors. In this respect, the International Self-Care Foundation [ISF] (2023a) presents SC as a set of person-centered activities that all citizens should carry out to maintain their health and well-being. This foundation emphasizes the great potential of making SC clearer, increasing its role in personal, family and community health.

The phenomenon of SC encompasses the domains: cognitive, psychosocial, physical, and behavioral (Matarese et al., 2019; Riegel et al., 2021). Each of these areas is interrelated and involved within the family. Through SC behaviors, which involve individual capacity, opportunity and motivation, people and families can and should be healthier, and remain so, managing their illnesses more effectively. Based on these assumptions, ISF (2023b) developed a framework for SC around seven pillars, which support a wide range of interconnected activities, covering the mentioned above cognitive, psychological, behavioral, and physical aspects. These, focus on health knowledge and literacy, mental well-being, physical activity, healthy eating, risk prevention, good hygiene, and the rational and responsible use of products and services. They also reflect the current perspective of SC, in particular the focus on understanding the processes that families experience, enabling them to achieve or maintain health and well-being (ISF, 2023b).

The FSC can thus be assumed as a complex union of domains that guide the family to be, behaving and becoming autonomous in the SC (Gray, 1996). There is consensus that this concept refers to an activity started, consciously, appropriate to the situation and focused on a goal (Riegel et al., 2021). And it is widely used in contexts of chronic diseases, given the context of acute diseases (Riegel et al., 2012, 2019; Tulu et al., 2021), based on three fundamental principles: autonomy, independence, and responsibility. These, understood as a process of health and well-being, innate but also learned (Riegel et al., 2021; Tulu et al., 2021). It can, similarly, be understood as the focus and result of health promotion and interventions to manage the disease aimed at improving the physical, psychosocial, and overall health condition of individuals (Riegel et al., 2021; Matarese et al., 2018). It has also been associated with broader notions of autonomy and responsibility (Martínez et al., 2021), reflecting the effective participation of the person/family in their health condition. Also in this perspective, WHO (2022) recently updated the definition of SC, transmitting it as the ability of individuals, families, and communities to promote health, prevent disease, and maintain health, even with diseases and/or deficits, adding that it can be developed with or without the support of a healthcare professional. In this assumption, it is found that the attitude of people has been changing, as in other areas of social life, with a growing tendency to be an integral part of the decision-making processes under preventive and treatment measures (Miller & Reihlen, 2023). This participation necessarily requires appropriation of relevant information and ability to decide with confidence and autonomy in health, which corroborates the learning aspect of SC through an efficient teaching, individualized and adapted to the needs and objectives of each person/family (Matthys et al., 2023). Therefore, a family-centered approach recognizes the strengths of individuals as active agents, and not merely passive recipients of health services (ISF, 2023c). And, for this to happen, we are fully aware that there are a wide variety of people who contribute to SC: care partners, family, healthcare professionals, home care providers, among others (Riegel et al., 2021). Of these, families will undoubtedly be a fundamental and primordial partner, due to its characteristics, favoring SC practiced at home, that is, in the normal context of family's daily lives (WHO, 2019).

## From Family Systemic Conception to Family Self-Care

The family systemic conception gained prominence, seen as an open sociocultural system, continually confronted with requirements to change both internally and externally (Gray, 1996). Indeed, within the family context, people must learn to adapt to the demands and stress (Olabisi et al., 2023), and it is in this perspective that the family system distinguishes and carries out its affective and socialization functions through subsystems, which can be individual, relational, or interpersonal.

Family members are linked in important ways through each stage of life, and these relationships are an important source of social connection and social influence for individuals throughout their lives (Hockenberry & Wilson, 2018; Thomas et al., 2017). Substantial evidence consistently shows that social relationships can profoundly influence well-being across the life course (Hockenberry & Wilson, 2018). It should also be recognized that the transformations that each family goes through lead to change, and, to understand this change, it is essential to identify its effects and meanings. These may be inherent to critical events or imbalances, which lead to changes in the ideals, perceptions, identities, relationships, and routines of the person and family (Zhan et al., 2022). Emphasizing this evidence, the theory of symbolic interactionism assigns meaning as one of the most important elements for understanding the human behavior, interactions, and processes, reaffirming that the meaning is a social product, that is, a creation that emanates from the activities of individuals as they interact (Meltzer et al., 2020). Thus, through Blumer's conception, healthcare professionals must be able to interact actively with families and must see things from their point of view and in their natural context (Blumer, 1986).

In this thread, the family translates a complex network of relationships and emotions, seen as a social unit (International Council of Nurses, 2012) or a collective whole, composed of members connected through consanguinity, emotional affinity, or legal kinship, including people who are important to the person being cared for. The person is no longer seen as the cause and the explanation of their possible problem, leaving the family with the explanatory responsibility for this observed (dys)functioning (Seltzer, 2019).

The currents dedicated to the analysis and study of the family, based on epistemological models and theories, particularly of systemic influence (Wright & Leahey, 2012), converge in the sense of clearly considering it a “being” one. The family is thus understood as a system, a globality that only in this holistic perspective can be correctly understood. Assuming systemic thinking as an epistemological reference that supports the understanding and interpretation of complexity and intersubjectivity inherent in the family, it seems relevant to invoke the systems general theory of von Bertalanffy’s (von Bertalanffy, 2015), which assumes a continuous interaction between the many members of the family system with the environment, assuming that the change in any “part” of the system affects all other “parts” (circular causality). It dares to affirm that the family understood as a system is not a sum of people inserted in a certain space or time but a group co-responsible for the well-being of all its members, so that everyone feels like an integral part of this whole. No matter what kind of family we face, they are all organized through a relational structure where roles and functions analogous to social expectations are defined. In this conjecture, families have the primary responsibility to meet the health needs of their members, and for this, it is essential that they have resources, opportunities, and well-being (Gray, 1996; Matthys et al., 2023). Thus, a pattern of FSC can be particularly relevant in specific contexts of the family life cycle, especially when there are children, who, due to their specific characteristics, are unable to SC. In this context, the family assumes a set of tasks and functions aimed at ensuring the survival, development, and well-being of children (Hockenberry & Wilson, 2018). These tasks and functions are performed throughout the life of children, in disparate conjunctures, but essentially in the family context, especially by parents, depending on their personal characteristics, family dynamics, and on environmental factors that surround them, providing conditions for health protection, maintenance, and promotion (Sanders & Mazzucchelli, 2017). Thus, parenthood, conceived as an elementary and essential dimension of the condition of human beings, is preliminarily the process by which a generation transmits to the next the values, skills, and attitudes necessary for survival as a species. Parents/family are responsible for a set of tasks that society expects to be fulfilled, ensuring not only their survival and safety but also a development as harmonious as possible (Hockenberry & Wilson, 2018; Sanders & Mazzucchelli, 2017).

The impact that a deficit or disorder has on the family may be dissimilar, considering its uniqueness; however, the literature states that this family will also adjust to the “different” situation of life (Hockenberry & Wilson, 2018). When a disorder results in severe damage to the adaptive process, the person cannot achieve standards of personal independence and social responsibility in one or more aspects of everyday life. Therefore, this condition admittedly chronic, gives the family increased tasks, responsibilities, and concerns (Beckmeyer & Russell, 2022).

As the family begins to know their health needs (individual and collective), it often becomes expert in promoting care, aiming to maintain, monitor, and manage SC effectively. Healthcare professionals, particularly nurses, are adjuncts and facilitators in this process, forming partnerships with the family, and when professional intervention is required (Venechuk et al., 2023). As such, professionals should assess the patient's family dynamics by collecting their social history and surroundings before developing an action plan. To help the family to develop and maintain a healthy family dynamics, mainly nurses can suggest intervention programs such as family sculpture and family therapy (Jabbari et al., 2023). Communication and effective negotiation between family and healthcare professionals are also essential to meet the management of chronic disease (Hockenberry & Wilson, 2018; Riegel et al., 2012, 2021). The family-centered care aims to assist the adaptation of the family throughout the trajectory of chronic disease, in its various changes, promoting the skills inherent to reach SC. Extending this approach, and taking as an example the family with a child with chronic disease, Riegel et al. (2012), through the middle range theory of self-care of chronic disease, attach to SC the essence of the management disease, defining it as a process of maintaining health through health promotion practices and disease management.

This theory divides the process into three interrelated elements, which make perfect sense in a familiar approach: self-care maintenance, self-care monitoring and self-care management. In this framework, the family manifests the ability to SC: being a whole, behaving through a process of learning and prior adaptation, and becoming autonomous in the various domains of SC. Research centered on life with chronic illness emphasizes the need to focus on family relationships and dynamics so that services can reduce suffering and promote healthy family functioning (Esandi et al., 2021). In this sense, FSC as a framework for the discipline of Nursing can be defined as a specific approach to clinical practice that recognizes the singularity and the family as a system, emphasizing its ability to promote, maintain, and protect health.

**Figure 1.**
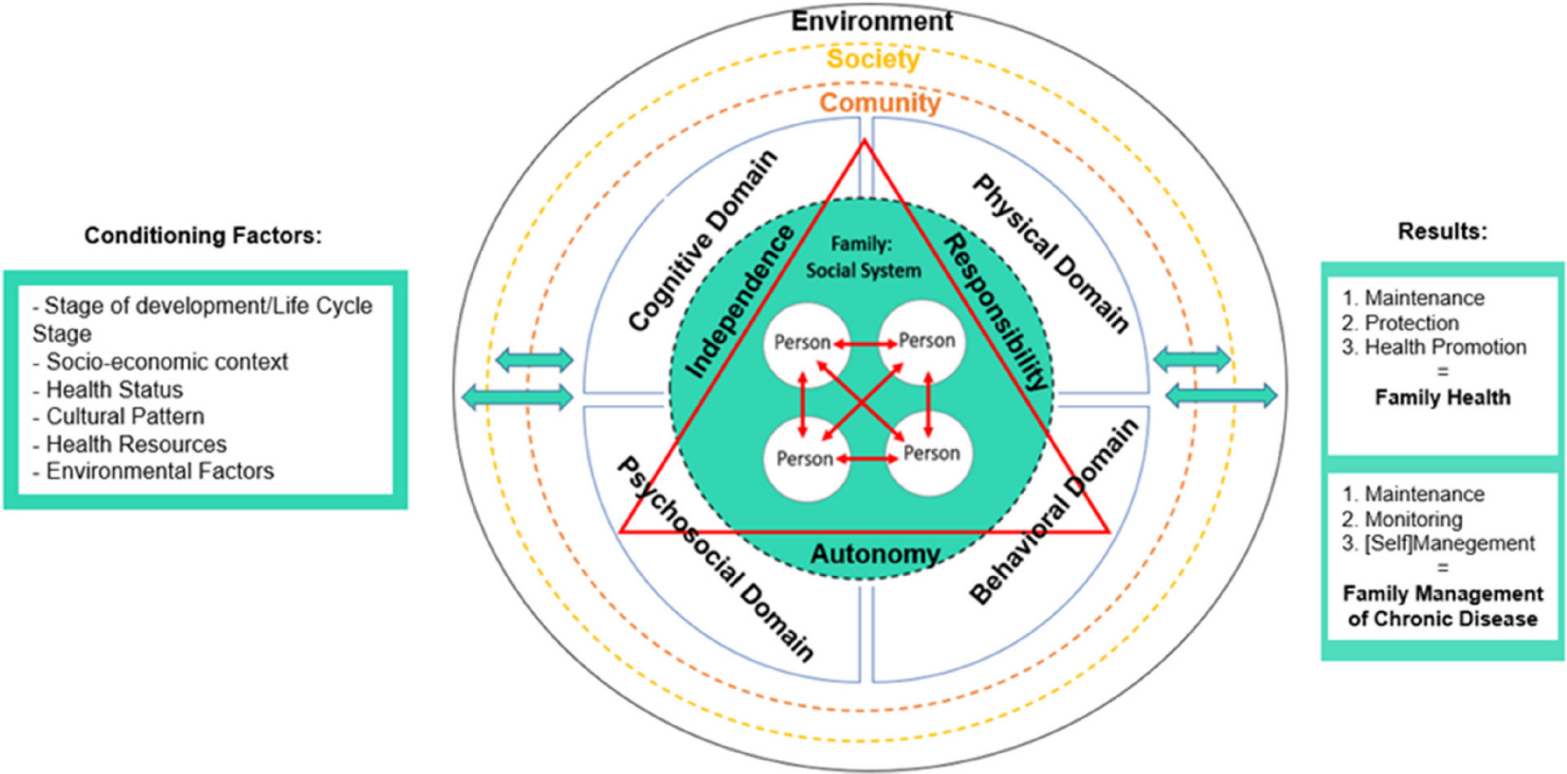
Family self-care—conceptual synthesis.

It is thus observed that FSC can be identified in any family, regardless of its characteristics. Because, in terms of outcomes, they all seek to achieve family health, whether in the context of chronic disease or not, maintaining health through health promotion and disease management practices, always mediated by FSC behaviors that they are supposed to develop. Accepting this definition, the decision to engage in SC behaviors and the effectiveness of the inherent actions will always be the responsibility of the family. In this sense, and to schematize the development of FSC, a conceptual synthesis was made in the form of a diagram (Figure 1).

Nevertheless, the development of FSC requires the systematic development of evidence, theory, and practice. Until now, because it is substantially outside the scope of current social and health systems, there has not been sufficient research and development about this subject in academia. There is a lack of a definitive canon of evidence justifying the absolute need for SC and the best ways to implement it in real family contexts (ISF, 2023c).

## Conclusions

The origin of SC appears in the family context despite being seen as a relevant determinant of individual health. It has become evident that the transformations that every family goes through trigger change, and to understand this change is fundamental to identify its effects and meanings.

In a context of families with inherent chronic disease, the pattern of FSC can assume a significant relevance in the sense of maintaining life, health, and well-being, demonstrating that the requirements of SC exceed individual capacity, requiring the cooperation of others. The evidence shows that the family transforms through times adapting and restructuring itself to continue functioning. Undoubtedly, we live in different groups (e.g., family, neighbors, work) and environments, in which we interact and develop. Although the most diverse social groups influence the life of each one of us, the family emerges as the most significant social group, assuming a socializing function, facilitating the integration of the individual in society, converting in a bridge between the individual and the collective.

As well, assessing and addressing family dynamics and its role in health and disease requires an interprofessional team of healthcare professionals, including nurses, physicians, social workers, and therapists. Nurses are uniquely positioned to observe interaction patterns, assess family relationships, and meet family concerns in the clinical setting since they frequently contact family members.

It was understood that FSC can be identified in any family, regardless of its characteristics. And as results, all seek to achieve family health, whether in the context of chronic disease or not, maintaining health through health promotion practices and disease management, always mediated by FSC behaviors, which are assumed to develop.

It was intended that this theme would contribute to increase knowledge, particularly in nursing, about how FSC develops and perpetuates itself, in the context of family health and self-management of chronic disease, allowing an early and systematic reflection on its importance for this discipline. Intervening in this perspective means perceiving the structure, functioning and development in relation to the family health-disease process. Therefore, this analysis allowed us to perceive the importance of ascertaining whether the family has an environment endowed with resources, which allows it to develop FSC behaviors, or whether, on the contrary, it constitutes a health problem.

## Practice Implications

Our findings suggest the pertinence of attention by healthcare professionals to the systemic conception of the family on the efficient management of their illnesses. It is understood that families with chronic diseases should be actively involved in SC and that its achievement will improve well-being, decrease morbidity and mortality, and reduce the costs associated with health care.

A FSC pattern may have a significant relevance in maintaining life, health status and well-being, showing that the demands of SC exceed the individual's capacity, requiring the cooperation of others, preferably performed within the family and home context. Once the prevalence of chronic diseases leads the population context, nurses and other healthcare professionals need to have knowledge that allows them, in an integrated way, to approach, care for, assist and teach the family as a fundamental unit of society, in the acquisition and maintenance of the self-care. Collaboration among the interprofessional healthcare teams can advance family-centered care practices, FSC and provide families with the necessary resources to develop and maintain healthy family dynamics.

Given the many challenges facing our vision, it is essential to further raise the profile of, and defend, SC as a vital element of health and healthcare. A definitive statement on the benefits and reasons for encouraging SC and making it part of official government policies remains to be made. The way forward seems to point to the implementation of solid, holistic policies aimed at promoting individual SC and FSC, changing professional practices or reorienting health systems towards a strategy of promotion, prevention, and SC.
